# The Diagnostic Accuracy of Urine-Based Xpert MTB/RIF in HIV-Infected Hospitalized Patients Who Are Smear-Negative or Sputum Scarce

**DOI:** 10.1371/journal.pone.0039966

**Published:** 2012-07-09

**Authors:** Jonathan G. Peter, Grant Theron, Tapuwa E. Muchinga, Ureshnie Govender, Keertan Dheda

**Affiliations:** 1 Lung Infection and Immunity Unit, Division of Pulmonology & UCT Lung Institute, Department of Medicine, University of Cape Town, Cape Town, South Africa; 2 Institute of Infectious Diseases and Molecular Medicine, University of Cape Town, Cape Town, South Africa; 3 Department of Infection, University College London Medical School, London, United Kingdom; National Institute for Infectious Diseases (L. Spallanzani), Italy

## Abstract

**Background:**

Hospitals in sub-Saharan Africa are inundated with HIV-infected patients and tuberculosis (TB) is the commonest opportunistic infection in this sub-group. Up to one third of TB-HIV co-infected patients fail to produce a sputum sample (sputum scarce) and diagnosis is thus often delayed or missed. We investigated the sensitivity of urine-based methods (Xpert MTB/RIF, LAM strip test and LAM ELISA) in such patients.

**Methodology/Principal Findings:**

281 HIV-infected hospitalised patients with clinically suspected TB provided a spot urine sample. The reference standard was culture positivity for *Mycobacterium tuberculosis* on ≥1 sputum or extra-pulmonary sample. MTB/RIF was performed using 1 ml of both unprocessed and, when possible, concentrated urine. Each unconcentrated urine sample was also tested using the Clearview LAM ELISA and Alere LAM strip test. 42% (116/242) of patients had culture-proven TB. 18% (20/54) were sputum scarce. In sputum-scarce patients, the sensitivity of urine MTB/RIF and LAM ELISA was 40% (95%CI: 22–61) and 60% (95%CI: 39–78), respectively. Urine MTB/RIF specificity was 98% (95%CI: 95–100). Combined sensitivity of urine LAM ELISA and MTB/RIF was better than MTB/RIF alone [MTB/RIF and LAM: 70% (95%CI: 48–85) vs. MTB/RIF: 40% (95%CI: 22–61), p = 0.03]. Significant predictors of urine MTB/RIF positivity were CD4<50 cells/ml (p = 0.001), elevated protein-to-creatinine ratio (p<0.001) and LAM ELISA positivity (p<0.001). Urine centrifugation and pelleting significantly increased the sensitivity of MTB/RIF over unprocessed urine in paired samples [42% (95%CI: 26–58) vs. 8% (95%CI: 0–16), p<0.001]. Urine MTB/RIF-generated C_T_ values correlated poorly with markers of bacillary burden (smear grade and time-to-positivity).

**Conclusions/Significance:**

This preliminary study indicates that urine-based MTB/RIF, alone or in combination with LAM antigen detection, may potentially aid the diagnosis of TB in HIV-infected patients with advanced immunosuppression when sputum-based diagnosis is not possible. Concentration of urine prior to MTB/RIF-testing significantly improves sensitivity.

## Introduction

In Africa, up to 65% of active tuberculosis (TB) cases are co-infected with HIV [1]. TB-related mortality is highest in this patient sub-group, and district-level hospitals are inundated with patients with advanced immunosuppression. With advancing HIV-related immunosuppression, the frequency of extra-pulmonary (EPTB) and disseminated forms of TB disease increase [2], [3], sputum smear microscopy performance is reduced, and up to a third of patients are unable to produce sputum for diagnostic testing [4]. Diagnosis is therefore challenging and often delayed, and post-mortem studies reveal a large burden of undiagnosed TB in HIV-infected hospitalised patients [5], [6], [7]. Recent studies have indicated that the rapid initiation of anti-TB treatment may reduce mortality [8]. There is a clear need for new, accurate, and rapid TB diagnostics that have utility in patients who cannot produce sputum.

The Clearview TB LAM ELISA (Alere Medical innovations, USA) detects LAM antigen in the urine and has recently evolved into a new point-of-care lateral flow test (Alere Determine-TB LAM Ag strip test) [9]. We recently found that this assay offered the greatest benefit in hospitalised HIV co-infected patients with advanced immunosuppression [4]. By contrast, the MTB/RIF assay is a novel, automated molecular TB diagnostic able to detect both the presence of *Mycobacterium tuberculosis* complex DNA and rifampicin drug-resistance in less than two hours. This test has been endorsed by the World Health Organization and is being rolled out in South Africa as a frontline test for individuals with suspected TB [10], [11]. Given the high accuracy of this test in sputum samples (sensitivity and specificity of 90% and 99%) [12], it represents a considerable advance over smear microscopy for the diagnosis of pulmonary-TB. However, acquiring a diagnostic sample remains a major hurdle in HIV-infected sputum scarce patients suspected of having active TB. Sputum induction, using ultrasonic nebulisation, may facilitate obtaining sputum, but this is often unavailable in hospitals in resource-poor settings and infection control is a concern. Tissue biopsies and aspirated samples may be obtained from extra-pulmonary disease foci (e.g. bone marrow and liver, pleural and pericardial fluid) but specialised skill and equipment requirements limit the availability and affordability in resource-poor settings. Urine is easily obtainable from sputum scarce patients but there are few data about the performance of newer diagnostic tests using urine [13].

We hypothesised that urine MTB/RIF may offer diagnostic utility in patients where sputum-based diagnosis is not feasible. The performance of this test specifically in sputum scarce patients with HIV has not been previously evaluated using urine.

## Methods

### Study Population

**Figure 1:**
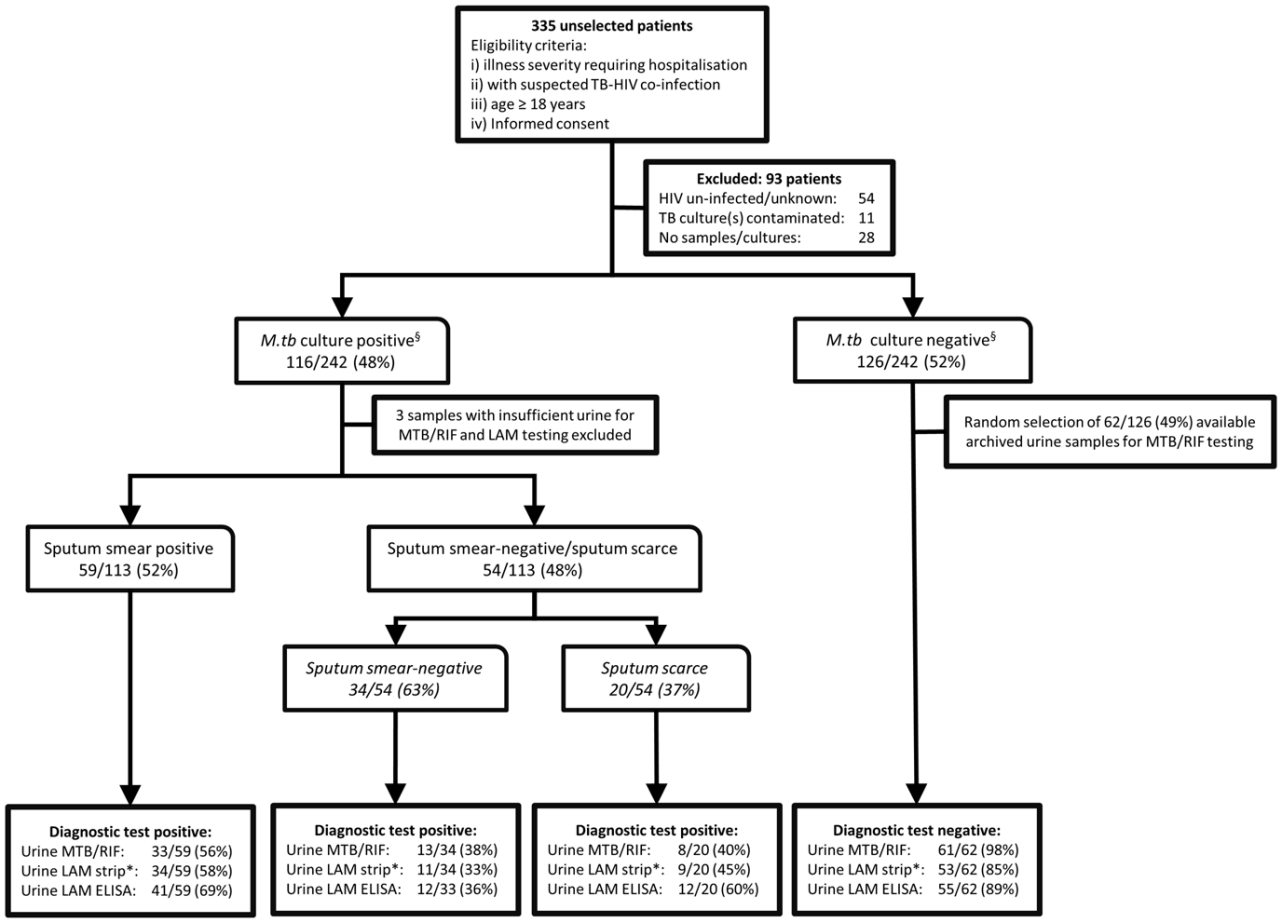
Study flow diagram. ^§^Both sputum and non-sputum samples (e.g. blood, pleural or pericardial fluid) were collected by attending clinicians for liquid TB culture. *M.tb* culture positive patients had at least one sputum or non-sputum sample liquid culture positive, while *m.tb* culture negative patients had at least one (usually 2 or more) samples liquid culture negative *Results using a grade 2 cut-point for the urine LAM strip test are shown.

The study population consisted of 335 prospectively recruited adult patients from four hospitals (three district- and one tertiary-level) between July 2009 and December 2010 in Cape Town, South Africa. Patients were referred for study inclusion by attending clinicians if the patient was suspected to have HIV-TB co-infection. Only three patients, who refused consent, were excluded from study enrolment. All other patients provided written informed consent and the study was approved by the University of Cape Town Faculty of Health Sciences Human Research Ethics Committee. Clinical information documented for enrolled patients included demographic information, past history of TB, co-morbidity, symptoms and vital signs, HIV status and renal function. A study outline is shown in Figure 1.

### Diagnostic Sample Collection Handling

Consultant-led groups of attending clinicians with no association to the study team decided on the timing and extent of diagnostic work-up, commencement of empiric anti-TB treatment, and final discharge from hospital. TB diagnostic work-up was not standardised, but routine local hospital practice includes the collection of two sputum samples in patients able to expectorate and, if EPTB is suspected, the collection of 1–2 non-sputum samples from clinically involved sites (e.g. fine needle aspirate of lymph node, pleural fluid aspirate/biopsy, ascitic tap, lumbar puncture, pericardial aspiration etc.). Further details of biological samples collected for TB culture are provided in table 1. The local reference laboratory processed all clinical specimens collected for TB diagnosis. Fluorescence smear microscopy was performed on NALC/NaOH processed sputum, which was also cultured using the MGIT 960 liquid culture system (BD Diagnostics, USA). The reference standard for definite-TB was liquid culture positivity for *M. tuberculosis*.

**Table 1:** Demographic, clinical and microbiological characteristics of *m.tb* culture positive and HIV-infected patients in the study population stratified by sputum smear microscopy and urine MTB/RIF results.

Patientcharacteristic(s)	≥1 sputum or non-sputum *m.tb* culture positive	Sputum smear positive	Sputum smear-negative or sputum scarce	Urine MTB/RIF positive, *m.tb* culture positive	Urine MTB/RIF negative, *m.tb* culture positive	P-value†
	(N = 113)	(N = 59)	(N = 54)	(N = 54)	(N = 59)	
	n (%)	n (%)	n (%)	n (%)	n (%)	
Age (years)	35	34	35	34	35	n/s
(median, IQR)	(28–38)	(27–38)	(28–38)	(28–39)	(28–38)	
Male	46 (41)	22 (37)	24 (44)	27 (50)	19 (32)	n/s
CD4 cell count#	89	109	80	56*	142*	*0.001
(median, IQR)	(45–198)	(50–215)	(40–167)	(33–134)	(59–241)	
Previous TB	32 (28)	19 (32)	13 (24)	13 (24)	19 (32)	n/s
Current Smoker	25 (22)	15 (25)	10 (19)	13 (24)	12 (20)	n/s
**Clinical features**						
Cough >2 wks	96 (85)	52 (88)	43 (80)	46 (85)	50 (85)	n/s
Night sweats	81 (72)	45 (76)	36 (67)	42 (78)	39 (66)	n/s
Weight loss	102 (90)	53 (88)	50 (93)	52 (96)*	50 (85)*	*0.003
Fever >38°C	29 (26)	19 (32)	10 (19)	16 (30)	13 (22)	n/s
**Clinical samples collected for TB culture**						
1 sputum sample	90 (80)	59 (100)*	34 (63)*	44 (82)	44 (78)	*<0.001
≥2 sputum samples	41 (36)	29 (49)*	12 (22)*	24 (44)	17 (29)	*0.003
1 non-sputum sample	75 (67)	29 (49)*	46 (85)*	35 (65)	40 (68)	*<0.001
≥2 non-sputum sample	25 (22)	7 (12)*	18 (33)*	15 (28)	10 (17)	*0.006
**Microbiological TB diagnosis**						
1× sputum *m.tb* culture positive	87 (77)	59 (100)*	28 (52)*	42 (78)	45 (76)	*<0.001
1× non-sputum culture *m.tb* positive	45 (40)§	14 (24)*	31 (57)*	24 (44)	21 (36)	*<0.001
1× both sputum & non-sputum *m.tb* culture positive	19 (17)	14 (24)*	5 (9)*	12 (22)	7 (12)	*0.04

# 9 patients did not have data CD4 count data.

§ Includes the following *m.tb* culture positive samples: 7 blood cultures, 11 pleural fluid samples, 4 pericardial fluid samples, 3 ascitic fluid samples, 8 cerebrospinal fluid (CSF) samples, 1 hip biopsy, 2 Lymph node biopsies, 5 fine needle aspirate, 1 gastric washing, 1 faeces and 2 pus swabs.

† P-values indicate significant differences between patient groups (marked with ^*^ to indicate comparison group) for demographic, clinical or microbiological characteristics.

### Urine Sampling and LAM Methodology

All patients were required to give a spot urine sample (10–30 ml) collected in a sterile container as soon as possible after recruitment. A urine dipstick test (*Uri*CHECK 9, RapiMed Diagnostics, South Africa) was immediately performed to assess for protein, blood and leucocytes, while the local reference laboratory performed urinary protein and creatinine measurements. Urine was frozen on the day of collection and stored at −20°C for later batched testing. Both the LAM ELISA and LAM strip test (a single manufacturing lot#101102) were performed on thawed urine according to the manufacturers’ instructions by readers blinded to patient data. Detailed methodology for both tests has been previously described [14]. Of note is the use of the grade 2 cut-point and not the manufacturer’s suggested grade 1 cut-point for the LAM strip test. This cut-point optimises test specificity and rule-in value [4] in hospitalised patients with HIV.

### Urine MTB/RIF Methodology

All HIV-infected patients with culture positive TB had an MTB/RIF performed using 1 ml of unprocessed, thawed urine according to the manufacturers’ suggested procedure for sputum samples [15]. In addition, a random sample of ∼50% (62/126) of culture negative non-TB patients had a urine MTB/RIF performed. The MTB/RIF operator was blinded to the clinical status of these patients. Briefly, the sample reagent was mixed at a 2∶1 ratio with ∼1 ml of urine. Two millilitres of the reagent sample mix was transferred into an MTB/RIF assay cartridge and inserted into the GeneXpert instrument [15]. Additionally, if the MTB/RIF was negative using a 1 ml urine sample, a second pelleted urine MTB/RIF was performed, where possible, using a median (IQR) of 10 (5–10) ml urine. Urine was centrifuged at 3000 *g* for 15 minutes and the pellet re-suspended in 1 ml of sterile phosphate-buffered saline. In culture-negative non-TB urine samples used for MTB/RIF, pelleting of up to 10 mls was performed where possible.

### Statistical Analysis

Sensitivity and specificity measures for all diagnostic tests are presented with 95% confidence intervals. Demographic, clinical and microbiological characteristics of different patient sub-groups were compared using χ2 and Wilcoxon rank-sum test as appropriate. Diagnostic sensitivity and specificity of individual and/or combinations of tests was compared using the χ2 and Fisher’s exact tests, as appropriate. Logistic regression analysis was used to identify predictors of urine MTB/RIF positivity (in all patients and restricted to smear-negative and sputum scarce patients). Spearman correlation (R_s_) was used to evaluate relationship between MTB/RIF-generated C_T_ values and other markers of bacillary load. All statistical tests were 2 sided at α = 0.05. STATA IC, version 10 (Stata Corp, Texas, USA) was used for all statistical analyses. Study reporting and analysis were consistent with the STARD criteria [16].

## Results

### Study Population and Proportion of Sputum Scarce Patients

Figure 1 outlines the study population and test results. 93 patients were excluded from the primary analysis, leaving 242 patients with ≥1 sputum or non-sputum (or both) liquid culture result. A further three patients had insufficient urine for MTB/RIF, LAM ELISA and strip testing. Patient demographic and clinical characteristics stratified by sputum smear status and urine MTB/RIF results are shown in Table 1. Additionally, table 1 shows details of all biological samples undergoing mycobacterial liquid culture. 48% (116/242) of included patients had culture-positive TB from either a sputum (n = 68), non-sputum sample (n = 26), or both (n = 22). Only 1/113 of culture positive patients had a positive urinary *m.tb* culture.

17% (42/242) of all patients with valid culture results and 18% (20/113) of culture positive were unable to produce sputum (sputum scarce). 52% (59/113) of TB patients were sputum smear-positive.

### Urine MTB/RIF Diagnostic Accuracy

Table 2 outlines the diagnostic accuracy of sputum smear microscopy, urine MTB/RIF, LAM ELISA and LAM strip test in all culture-positive patients stratified by CD4 cell count and sputum scarce culture-positive patients only. Overall, MTB/RIF had a sensitivity of 48% (95% CI: 39–57; 54/113), equivalent to the overall sensitivities of sputum smear microscopy [52% (95% CI: 43–61; 59/113)], urine LAM ELISA [58% (95% CI: 49–67; 65/112)] and LAM strip test (grade 2 cut-point) [48% (95% CI: 39–57; 55/113)]. Urine MTB/RIF sensitivity was higher in patients with CD4≤200 cells/ml vs. CD4≥200 cells/ml [54% (95% CI: 43–65; 42/78) vs. 31% (95% CI: 17–50; 8/26), p = 0.04]. The highest urine MTB/RIF sensitivity of 61% (95% CI: 48–73; 33/54) was in CD4≤100 cells/ml. In sputum scarce, non-sputum culture-positive patients, the sensitivity of urine MTB/RIF was 40% (95%CI: 22–61; 8/20). Additionally, one of the eight urine MTB/RIF positive patients was found to have rifampicin-resistance and this was confirmed by phenotypic drug-susceptibility testing. Urine MTB/RIF sensitivity was equivalent to urine LAM ELISA and LAM strip test regardless of a patient’s ability to produce sputum or sputum smear status. In sputum smear-negative patients, the sensitivity of urine MTB/RIF was 39% (95%CI: 27–52; 21/54). The specificity of urine-based MTB/RIF was 98% (95%CI: 95–100; 61/62), which was higher than both the urine LAM ELISA and strip test [98% (95%CI: 95–100) vs. 89% (95%CI: 81–97), p = 0.03].

**Table 2:** Diagnostic accuracy of sputum smear microscopy, urinary MTB/RIF, TB LAM ELISA, LAM strip test (grade 2 cut-point) and clinically relevant combinations thereof in any sputum/non-sputum *m.tb* culture positive patients overall, in sputum scarce patients only, and stratified by CD4 cell count†.

Diagnostic test(s)	All *m.tb* culture positive	Only sputum-scarce non-sputum *m.tb* culture positive	HIV-infected patients withCD4 count >200 cells/ml	HIV-infected patients withCD4 count ≤200 cells/ml	Random sample of *m.tb* culture negative patients§
	(N = 113)	(N = 20)	(N = 26)	(N = 78)	(N = 62)
	Sensitivity (%)	Sensitivity (%)	Sensitivity (%)	Sensitivity (%)	Specificity (%)
	(95% CI)	(95% CI)	(95% CI)	(95% CI)	(95% CI)
	n/N	n/N	n/N	n/N	n/N
**Sputum smear microscopy**	52^#3^		58^#6 #7^	50^#8^	100
	(43–61)	N/A	(39–75)	(39–61)	(94–100)
	59/113		15/26	39/78	62/62
**Urine MTB/RIF**	48^#1^	40^#5^	31^#7^ * ^1^	54^#9^ * ^1^	98^#11^
	(39–57)	(22–61)	(17–50)	(43–65)	(95–100)
	54/113	8/20	8/26	42/78	61/62
**Urine LAM ELISA**	58^#4^	60	27* ^2^	69^#8^ * ^2^	89^#11^
	(49–67)	(39–78)	(14–46)	(58–78)	(81–97)
	65/112	12/20	7/26	53/77	55/62
**Urine LAM strip test** **(grade 2 cut-point)**	48^#2^	45	27^#6^ * ^3^	56^#10^ * ^3^	85
	(39–57)	(26–66)	(14–46)	(45–67)	(77–94)
	55/113	9/20	7/26	44/78	53/62
**Urine LAM ELISA followed by** **urine MTB/RIF (performed if** **LAM ELISA negative)**	68^#1 #2^	70^#5^	38* ^4^	79^#9 #10^ * ^4^	89
	(60–77)	(48–85)	(20–57)	(71–88)	(81–97)
	77/113	14/20	10/26	62/78	55/62
**Urine LAM ELISA combined** **with smear microscopy**	74^#3 #4^		58* ^5^	80* ^5^	89
	(65–82)	N/A	(39–77)	(71–88)	(81–97)
	83/113		15/26	62/78	55/62

# Indicates p<0.05 for a comparison of the sensitivity between different tests (e.g. urine MTB/RIF vs. LAM strip test) or combinations thereof; specific p-value: ^#1^p = 0.002; ^#2^p = 0.003; ^#3^p = 0.001; ^#4^p = 0.01; ^#5^p = 0.06; ^#6^p = 0.02; ^#7^p = 0.05; ^#8^p = 0.02; ^#9^p<0.001; ^#10^p = 0.002; ^#11^p = 0.03.

* Indicates p<0.05 for a comparison of differences in sensitivity between CD4>200 and ≤200 groups for a specific test or combinations thereof; specific p-values: ^*1^p = 0.04;^ *2^p<0.001; ^*3^p = 0.009; ^*4^p<0.001; ^*5^p = 0.03; non-significant p-values not shown.

† 9 culture positive patients with no available CD4 cell count results.

§ Reference standard of culture was used which does not account for persons with culture-negative, clinical TB.

### The Effect of Urine Centrifugation on MTB/RIF Performance

33% (38/116) and 41% (25/61) of culture positive and negative patients respectively had sufficient archived urine available to perform MTB/RIF using both 1 ml unprocessed and 2–10 ml centrifuged and pelleted urine. The median (IQR) of urine used for pelleting was 10 (5–10) ml. Comparing paired samples, the sensitivity of urine MTB/RIF was higher using 2–10 ml centrifuged and pelleted urine than 1 ml unprocessed urine [42% (95%CI: 26–58; 16/38) vs. 8% (95%CI: 0–16; 3/38), p<0.001]. Specificity of urine MTB/RIF was not affected by pelleting. Pelleting allowed an additional 13 cases of TB to be detected, four of which were from sputum scarce patients. Centrifugation and pelleting of 6–10 mls produced a non-significant increase in sensitivity compared with 2–5 mls [48% (95%CI: 29–67; 13/27) vs. 28% (95%CI: 0–54; 3/11), p = 0.2]. No difference was noted in the mean (SD) MTB/RIF internal positive control C_T_-value for the MTB/RIF using 1 ml vs. 2–10 ml urine volumes [25.8 (1.4) vs. 25.9 (1.8), p = 0.4].

### Combined Urine LAM ELISA and MTB/RIF

**Figure 2:**
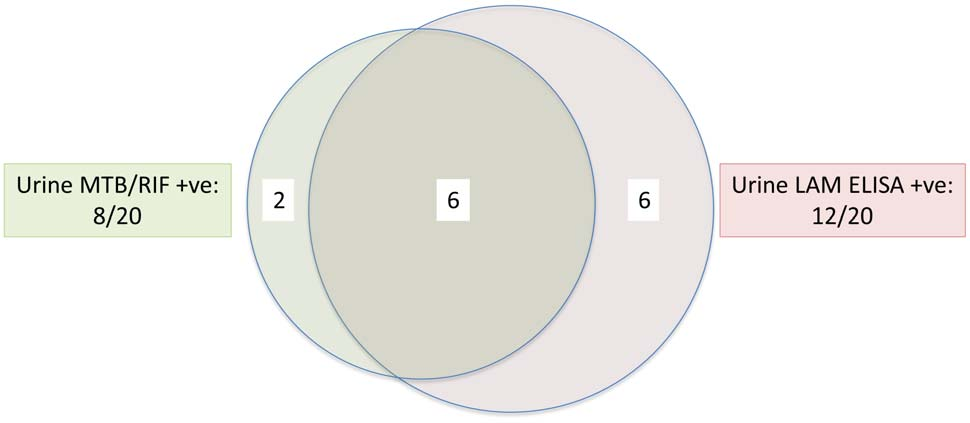
Venn diagram showing the proportions of patients diagnosed by urine MTB/RIF and/or urine LAM ELISA in sputum scarce *m.tb* culture positive patients. 6 patients were both urine MTB/RIF and LAM ELISA positive; 6 patients were urine LAM ELISA positive, but MTB/RIF negative, while 2 patients were urine MTB/RIF positive, LAM ELISA negative.

Figure 2 shows the proportions of sputum scarce TB patients that were test-positive for both the urine LAM ELISA and MTB/RIF or on only one of the two tests. Both urine MTB/RIF and LAM ELISA detected six cases, while urine MTB/RIF and LAM ELISA combined detected an additional two and six cases, respectively. Table 2 shows that the combined sensitivity in sputum scarce patients of urine LAM ELISA followed by MTB/RIF was 70% (95% CI: 48–85; 14/20), compared to 40% (95% CI: 22–61; 8/20) for urine MTB/RIF (p = 0.06), 60% (95% CI: 39–78; 12/20) for LAM ELISA (p = 0.5), and 45% (95%CI: 26–66, 9/20) for LAM strip test (p = 0.1) alone. Overall, and in other relevant patient sub-groups (sputum smear-negative/sputum scarce, CD4≤200 or ≤100 cells/ml), the combined sensitivity of urine LAM ELISA and MTB/RIF was better than urine MTB/RIF and LAM strip tests alone, but not urine LAM ELISA alone.

### Predictors of Urine MTB/RIF Test Positivity

Table 3 presents univariate associations of MTB/RIF positivity in HIV-infected culture-positive patients stratified by sputum smear status. CD4≤50 cells/ml, protein/creatinine ratio >0.03 g/l and urine LAM ELISA positivity were strong predictors of urine MTB/RIF positivity. Additionally, patients with a glomerular filtration rate (GFR) between 30–60 ml/min were more likely to have a positive MTB/RIF [OR (95%CI): 3.0 (1.0–8.3), p = 0.04]. Previous TB, smoking status, any TB symptom, admission vital signs and urine dipstick abnormalities were not associated with MTB/RIF positivity. In sputum smear/negative and sputum scarce patients, only a CD4≤50 and a protein/creatinine ratio >0.03 g/l were strong predictors of urine MTB/RIF positivity.

**Table 3:** Associates of MTB/RIF positivity in HIV-infected *m.tb* culture positive patients stratified by smear status.

Patient characteristic (s)	Odds ratio (95% CI)	P-value
**All patients (sputum smear positive and negative)**		
CD4≤200	2.6 (1.0–6.7)	0.05
CD4≤100	3.1 (1.4–6.8)	0.006
CD4≤50	5.3 (2.0–13.9)	0.001
Protein/creatinine ratio >0.03 g/l	6.2 (2.3–17.1)	<0.001
Urea≥7.1 mmol/l	1.6 (0.7–3.9)	0.3
GFR 30–60 ml/min*	3.0 (1.0–8.3)	0.04
Urine LAM ELISA	5.0 (2.2–11.4)	<0.001
**Sputum smear-negative/scarce patients only**		
Age (years)	1.08 (1.00–1.16)	0.04
CD4≤200	3.9 (0.7–20.2)	0.11
CD4≤100	5.2 (1.4–19.4)	0.01
CD4≤50	9.8 (2.4–38.8)	0.001
Protein/creatinine ratio >0.03 g/l	5.3 (1.3–21.8)	0.02
Urea≥7.1 mmol/l	1.3 (0.3–4.8)	0.3
GFR 30–60 ml/min*	2.4 (0.4–14.0)	0.3
Urine LAM ELISA	2.6 (0.9–8.2)	0.1

* Glomerular filtration rate calculated using the modified Cochrane-Gault equation.

### Urine MTB/RIF C_T_ Values and Relationship to Other Bacillary Burden Markers

**Figure 3:**
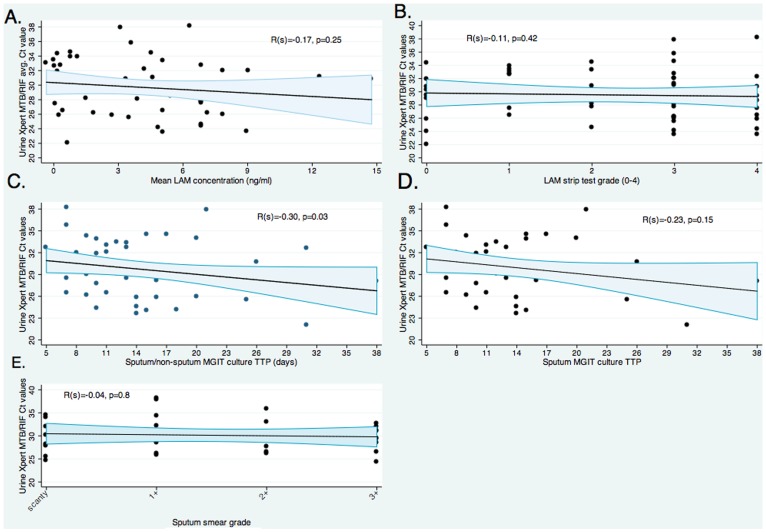
Urine-based MTB/RIF-generated C_T_ values correlates with other markers of bacillary burden. In *m.tb* culture and urine MTB/RIF positive patients, MTB/RIF-generated C_T_ values were correlated with (A) urine LAM ELISA concentration, (B) urine LAM strip grading, (C) either sputum or non-sputum liquid culture time-to-positivity (TTP), (D) only sputum liquid culture time-to-positivity, and (E) smear-grade in smear positive patients.

In urine MTB/RIF positive patients, the mean (sd) C_T_ -value was 21.3 (13.8). Two-way scatter plots in figure 3 explore correlations between urine C_T_ -values and other markers of bacillary burden. No correlations between urine MTB/RIF-generated C_T_ -values and mean urine LAM concentration [spearman rho: 0.17, p = 025], LAM strip test grade [spearman rho: 0.11, p = 0.4], sputum liquid culture time-to-positivity (TTP) [spearman rho: 0.23, p = 0.15] and/or sputum smear microscopy grade (in smear positive patients) [spearman rho: 0.04, p = 0.8] were found. No correlation between urine C_T_ -values and urine culture TTP was possible as only 1/113 urine cultures was positive. A weak, but significant inverse relationship between urine C_T_ -value and liquid culture TTP [spearman rho: 0.3, p = 0.03] was noted. Additionally, urine MTB/RIF-generated CT-values showed no significant correlation with CD4 T-cell count [spearman rho: 0.27, p = 0.06].

## Discussion

Sputum scarce, smear-negative and EPTB constitute a major burden of undiagnosed TB in HIV co-infected hospitalised patients [5]. Misdiagnosis and diagnostic delays lengthen hospital stay and delay rapid treatment initiation, likely worsening TB-related morbidity and mortality [8]. Urine-based diagnostics may be particularly useful in these patients. The key findings of this preliminary study are: i) urine MTB/RIF can detect TB, and rifampicin resistance where applicable, in hospitalised HIV-infected patients who cannot produce a sputum sample (the assay detected 8 cases who would have been missed by conventional sputum-based diagnostics i.e. 7% of culture positive TB cases); ii) centrifugation and pelleting of 2–10 mls of urine significantly improved diagnostic yield vs. 1 ml unprocessed urine without impacting the MTB/RIF indeterminate rate (almost a third of patients diagnosed exclusively using centrifuged urine were sputum scarce); iii) in sputum scarce, and other patient sub-groups, the combination of urine LAM ELISA with urine MTB/RIF improved sensitivity compared with urine MTB/RIF or LAM strip tests alone; and iv) urine MTB/RIF positivity was strongly associated with advanced immunosuppression and proteinuria (suggesting a ‘leaky’ renal filtration mechanism).

Approximately 10–20% of HIV-infected patients with suspected TB are unable to produce sputum for sputum-based diagnostics [4], [17]. An additional 50–70% will have smear-negative active TB [18]. Sputum induction by ultrasonic nebulisation is an important sampling adjunct but availability is very limited in resource-poor settings, infection control is problematic, and sputum is still unobtainable in ∼10% of patients [17]. Thus, urine-based diagnostics have the potential to offer incremental diagnostic yield in this context. Urinary LAM, both as an ELISA-kit and recently as a lateral flow strip test, is the most extensively evaluated urine-based TB diagnostic [9], [13], [19]. It is most useful as a rule-in test in HIV-infected persons with advanced immunosuppression [4], [20], [21]. However, concerns about context-specific test specificity, optimal cut-point selection (for the LAM strip test), and lack of a drug-susceptibility read-out remain [4]. In this study we show the overlapping, yet non-redundant, performance of urinary LAM and MTB/RIF, and the improved overall diagnostic sensitivity of using the tests in combination. There is limited information about the performance of MTB/RIF in combination with adjunct diagnostics [22].

There are limited published data about the performance of MTB/RIF using urine samples. Hilleman *et al*., in a selected laboratory cohort interrogating extra-pulmonary samples found that MTB/RIF sensitivity was 100% in 6 culture-positive urine samples with unknown HIV status [23], while Lawn *et al*, in HIV-infected out-patients pre-ARV initiation, report the overall sensitivity of urine MTB/RIF to be 19% [24]. By contrast, our study focused on sputum scarce, hospitalised HIV-infected patients where diagnosis is often delayed and challenging. It is likely that HIV-infected patients’ with more advanced immunosuppression accounted for the higher urine MTB/RIF sensitivity found in our study.

We found a strong association between declining CD4 cell count, LAM in the urine, proteinuria, and increasing urine MTB/RIF positivity. This may reflect renal TB as part of disseminated TB, increased bacillary burden in those with the most advanced immunosuppression, a ‘leaky’ filtration mechanism or a combination of these. That a minority of the MTB/RIF-positive samples were urine culture positive may simply reflect sampling error, the limited volume sent for culture, or that there are several mechanisms driving urine test positivity. Further molecular biological and pathological studies are required to shed more light on the underlying mechanisms.

The sensitivity of urine MTB/RIF was markedly improved by the centrifugation and pelleting of ∼ 2–10 mls urine. Indeed, the concentration of a number of biological samples, such as cerebrospinal and pleural fluid, has improved the performance of traditional TB diagnostics [25]. However, concentration is also believed to increase PCR inhibition and potentially the rate of indeterminate test results. With only a single indeterminate test result (on a 1 ml urine sample), and no change in the mean internal positive control C_T_-value in centrifuged samples, we feel that 10 mls of centrifuged and pelleted urine is optimal when using MTB/RIF. The incremental yield of using volumes greater than 10 mls will require further evaluation.

Sputum-based MTB/RIF-generated C_T_-values have been shown to strongly correlate with other markers of bacillary load, such as liquid culture TTP and smear grade [15], [26], [27]. However, we could show no such correlation with urine (either with urine or sputum TTP and smear grade). This lack of correlation likely reflects limited sample numbers, and the differential relationship between urine bacillary load, renal abnormalities (renal TB, glomerular dysfunction etc.) and total body bacillary load.

This preliminary study has important limitations. No sputum-based MTB/RIF was performed in our study, thus we could not compare performance between urine and sputum. However, sputum samples were not stored in the parent study [4] and this caveat is redundant in sputum scarce patients, which is the very subgroup targeted by our study. Archived, frozen urine samples were used for all TB diagnostic tests. This may have affected the diagnostic performance of urine culture, but prior studies suggest no impact on the performance of MTB/RIF [28] or LAM [19] in this context.

In conclusion, this preliminary study indicates that urinary MTB/RIF may aid the rapid diagnosis of TB in sputum scarce HIV-infected patients with advanced immunosuppression. Moreover, urine centrifugation significantly improves sensitivity. Used alone, or in combination with urine LAM, urine-based MTB/RIF may potentially offer ∼70% of HIV co-infected persons a TB diagnosis with 24 hours of hospital admission. Finally, as demonstrated in this study, the ability of MTB/RIF to offer rifampicin drug susceptibility testing is an important advantage over urine LAM. Further prospective studies in larger cohorts using standardised TB diagnostic work-up are required to clarify these findings.
